# Slowness: An Objective for Spike-Timing–Dependent Plasticity?

**DOI:** 10.1371/journal.pcbi.0030112

**Published:** 2007-06-29

**Authors:** Henning Sprekeler, Christian Michaelis, Laurenz Wiskott

**Affiliations:** Institute for Theoretical Biology, Humboldt-Universität zu Berlin, Berlin, Germany; UFR Biomédicale de l'Université René Descart, France

## Abstract

Our nervous system can efficiently recognize objects in spite of changes in contextual variables such as perspective or lighting conditions. Several lines of research have proposed that this ability for invariant recognition is learned by exploiting the fact that object identities typically vary more slowly in time than contextual variables or noise. Here, we study the question of how this “temporal stability” or “slowness” approach can be implemented within the limits of biologically realistic spike-based learning rules. We first show that slow feature analysis, an algorithm that is based on slowness, can be implemented in linear continuous model neurons by means of a modified Hebbian learning rule. This approach provides a link to the trace rule, which is another implementation of slowness learning. Then, we show analytically that for linear Poisson neurons, slowness learning can be implemented by spike-timing–dependent plasticity (STDP) with a specific learning window. By studying the learning dynamics of STDP, we show that for functional interpretations of STDP, it is not the learning window alone that is relevant but rather the convolution of the learning window with the postsynaptic potential. We then derive STDP learning windows that implement slow feature analysis and the “trace rule.” The resulting learning windows are compatible with physiological data both in shape and timescale. Moreover, our analysis shows that the learning window can be split into two functionally different components that are sensitive to reversible and irreversible aspects of the input statistics, respectively. The theory indicates that irreversible input statistics are not in favor of stable weight distributions but may generate oscillatory weight dynamics. Our analysis offers a novel interpretation for the functional role of STDP in physiological neurons.

## Author Summary

Neurons interact by exchanging information via small connection sites, so-called synapses. Interestingly, the efficiency of synapses in transmitting neuronal signals is not static, but changes dynamically depending on the signals that the associated neurons emit. As neurons receive thousands of synaptic input signals, they can thus “choose” the input signals they are interested in by adjusting their synapses accordingly. This adaptation mechanism, known as synaptic plasticity, has long been hypothesized to form the neuronal correlate of learning. It raises a difficult question: what aspects of the input signals are the neurons interested in, given that the adaptation of the synapses follows a certain mechanistic rule? We address this question for spike-timing–dependent plasticity, a type of synaptic plasticity that has raised a lot of interest in the last decade. We show that under certain assumptions regarding neuronal information transmission, spike-timing–dependent plasticity focuses on aspects of the input signals that vary slowly in time. This relates spike-timing–dependent plasticity to a class of abstract learning rules that were previously proposed as a means of learning to recognize objects in spite of contextual changes such as size or position. Based on this link, we propose a novel functional interpretation of spike-timing–dependent plasticity.

## Introduction

The ability to recognize objects in spite of possible changes in position, lighting conditions, or perspective is doubtlessly an advantage in everyday life. However, our brain usually performs this task with such astonishing ease that we are seldom aware of the complexity this recognition problem comprises. On the level of primary sensory signals (e.g., light that stimulates a single retinal receptor), even small changes in the position of the object to be recognized may lead to vastly different stimuli. Our brain thus has to somehow identify rather different stimuli as representations of the same underlying cause, i.e., it has to develop an internal representation that is invariant to irrelevant changes of the stimulus. The work presented here is motivated by the question of how such invariant representations could be established.

Because of the limited amount of information in the genome as well as the apparent flexibility of the neural development in different environments, it seems unlikely that the information needed to form invariant representations is already there at the beginning of individual development. Some information must be gathered from the sensory input experienced during interaction with the environment; it has to be learned. As this learning process is likely to be at least partially unsupervised, the brain requires a heuristics as to what stimuli should be classified as being the same.

One possible indicator for stimuli to represent the same object is temporal proximity. A scene that the eye views is very unlikely to change completely from one moment to the next. Rather, there is a good chance that an object that can be seen now will also be present at the next instant of time. This implies that invariant representations should remain stable over time, that is, they should vary slowly. Inverting this reasoning, a sensory system that adapts to its sensory input in order to extract slowly varying aspects may succeed in learning invariant representations. This “slowness” or “temporal stability” principle is the basis of a whole class of learning algorithms [1–7]. Most applications of this approach have focused on models of the visual system, in particular on the self-organized formation of complex cell receptive fields in the primary visual cortex [8,9].

For clarity, we will focus on one of these algorithms, slow feature analysis (SFA; [10]); a close link to the so-called “trace rule” will arise naturally. The goal of SFA is the following: given a multidimensional input signal **x**(*t*) and a finite-dimensional function space F, find the input–output function *g*_1_(**x**) in F that generates the most slowly varying output signal *y*_1_(*t*) = *g*_1_(**x**(*t*)). It is important to note that the function *g*_1_(**x**) is required to be an instantaneous function of the input signal. Otherwise, slow output signals could be generated by low-pass filtering the input signal. As the goal of the slowness principle is to detect slowly varying features of the *input* signals, a mere low-pass filter would certainly generate slow output signals, but it would not serve the purpose.

As a measure of slowness, or rather “fastness,” SFA uses the variance of the time derivative,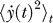 , which is the objective function to be minimized. Here, 〈·〉*_t_* denotes temporal averaging. For mathematical convenience and to avoid the trivial constant response, *y*_1_(*t*) = const, a zero-mean, and unit variance constraint are imposed. Furthermore, it is possible to find a second function *g*_2_(**x**) extracting *y*_2_(*t*) = *g*_2_(**x**(*t*)) that again minimizes the given objective under the constraint of being uncorrelated with *y*_1_(*t*), a third one uncorrelated with both *y*_1_(*t*) and *y*_2_(*t*), and so on, thereby generating a set of slow features of the input ordered by the degree of slowness. However, in this paper, we will consider just one single output unit.

SFA has been applied to the learning of translation, rotation, and other invariances in a model of the visual system [10], and it has been shown that when applied to image sequences generated from static natural images, SFA learns functions that reproduce a wide range of features of complex cells in primary visual cortex [8]. Iteration of the same principle in a hierarchical model in combination with a sparseness objective has been used to model the self-organized formation of spatial representations resembling place cells as found in the hippocampal formation of rodents [11] (see [12] for related work).

These findings suggest that on an abstract level SFA reflects certain aspects of cortical information processing. However, SFA as a technical algorithm is biologically rather implausible. There is in particular one step in its canonical formulation that seems especially odd compared with what neurons are normally thought to do. In this step the eigenvector that corresponds to the smallest eigenvalue of the covariance matrix of the time derivative of some multidimensional signal is extracted. The aim of this paper is to show how this kind of computation can be realized in a spiking model neuron.

In the following, we will first consider a continuous model neuron and demonstrate that a modified Hebbian learning rule enables the neuron to learn the slowest (in the sense of SFA) linear combination of its inputs. Apart from providing the basis for the analysis of the spiking model, this section reveals a mathematical link between SFA and the trace learning rule, another implementation of the slowness principle. We then examine if these findings also hold for a spiking model neuron, and find that for a linear Poisson neuron, spike-timing–dependent plasticity (STDP) can be interpreted as an implementation of the slowness principle.

## Results

### Continuous Model Neuron

#### Linear model neuron and basic assumptions.

First, consider a linear continuous model neuron with an input–output function given by

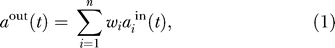
with 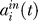 indicating the input signals, *w_i_* the weights, and *a* ^out^ the output signal. For mathematical convenience, let 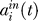 and *a* ^out^ (*t*) be defined on the interval *t* ∈ [−∞, ∞] but differ from zero only on [0,*T*], which could be the lifetime of the system. We assume that the input is approximately whitened on any sufficiently large interval [*t_a_*,*t_b_*] ⊂ [0,*T*] (i.e., each input signal has approximately zero mean and unit variance and is uncorrelated with other input signals):

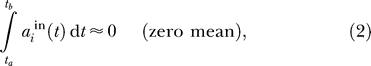


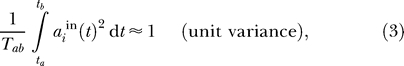


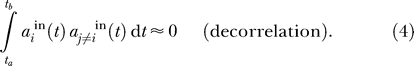
This can be achieved by a normalization and decorrelation step of the units projecting to the considered unit. Furthermore, we assume that the output is normalized to unit variance, which for whitened input means that the weight vector is normalized to length 1. In an online learning rule, this could be implemented by either an activity-dependent or a weight-dependent normalization term. Thus, for the output signal we have:

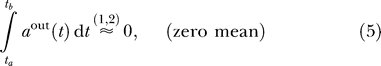


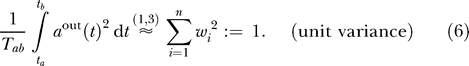
In the following, we will often consider filtered signals. Therefore, we introduce abbreviations for the convolution *f*_∘_ *g* and the cross-correlation *f* * *g* of two functions *f*(*t*) and *g*(*t*):

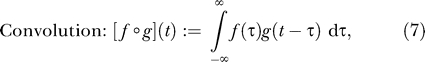


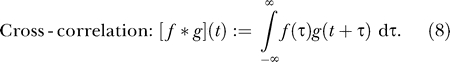
For convenience, we will often use windowed signals, indicated by a hat

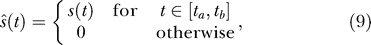
which allows us to replace the integration of a signal *s*(*t*) over [*t_a_*,*t_b_*] by an integration of ŝ*(t)* over [−∞, ∞]. We assume that the interval [*t_a_*,*t_b_*] is long compared to the width of the filters. In this case, effects from the integration boundaries are negligible, and we have

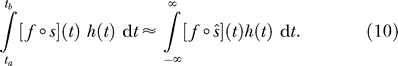
Similar considerations hold for the cross-correlation (Equation 8).

Since convolution and cross-correlation are conveniently treated in Fourier space, we repeat the definition of the Fourier transform 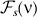 and the power spectrum *P_s_*(*ν*) of a signal *s*(*t*).

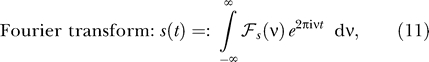


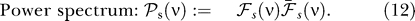



Throughout the paper, we make the assumption that input signals (and hence also the output signals) do not have significant power above some reasonable frequency *ν_max_*.

#### Reformulation of the slowness objective.

**Figure 1:**
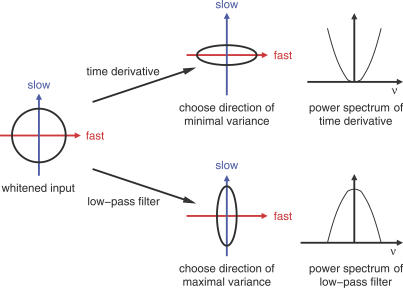
Choosing Slow Directions of the Input Finding the direction of least variance in the time derivative of the input (which is part of the SFA algorithm) can be replaced by finding the direction of maximum variance in an appropriately low-pass filtered version of the input signal.

SFA is based on the minimization of the second moment of the time derivative, 
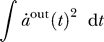
 . Even though there are neurons with transient responses to changes in the input, we believe it would be more plausible if we could derive an SFA-learning rule that does not depend on the time derivative, because it might be difficult to extract, especially for spiking neurons. It is indeed possible to replace the time derivative by a low-pass filtering as follows:

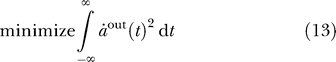


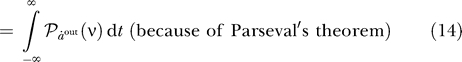


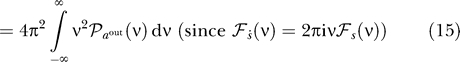


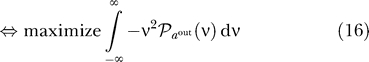


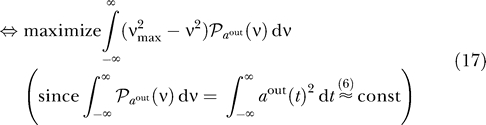


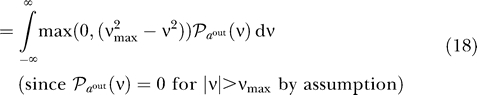


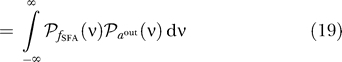


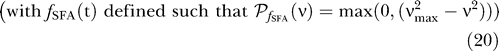


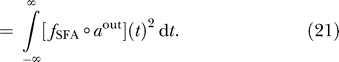
Thus, SFA can be achieved either by minimizing the variance of the time derivative of the output signal or by maximizing the variance of the appropriately filtered output signal. Figure 1 provides an intuition for this alternative. The filter *f_SFA_* is obviously a low-pass filter, as one would expect, with a 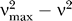 power spectrum below the limiting frequency *ν*_max_. Because the phases are not determined, further assumptions are required to fully determine an SFA filter. However, we will proceed without defining a concrete filter, since it is not required for the considerations below.

#### Hebbian learning on filtered signals.

It is known that standard Hebbian learning under the constraint of a unit weight vector applied to a linear unit maximizes the variance of the output signal. We have seen in the previous section that SFA can be reformulated as a maximization problem for the variance of the low-pass filtered output signal. To achieve this, we simply apply Hebbian learning to the filtered input and output signals, instead of to the original signals.

Consider a hypothetical unit that receives low-pass filtered inputs and, therefore, because of the linearity of the unit and the filtering, generates a low-pass filtered output

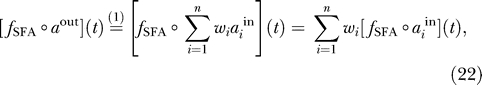
where *f_SFA_* is the kernel of the linear filter applied. It is obvious that a *filtered Hebbian learning rule*

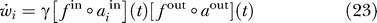
with *f* ^in^: = *f* ^out^: = *f_SFA_* maximizes the objective in Equation 21.

**Figure 2:**
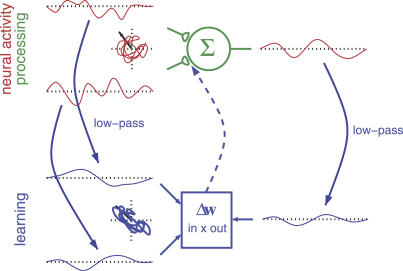
Filtered Hebbian Learning Rule Input and output signals are filtered (downward arrows). The weight change is the result of applying the Hebbian learning rule on the filtered signals (square box and upward arrow). Thereby, the variance of the filtered version of the output is maximized without actually filtering the output during processing.

Remember that the input is white (i.e., the 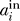 are uncorrelated and have unit variance), and the weight vector is normalized to norm one by some additional normalization rule, so that we know that the output signal *a* ^out^ has the same variance no matter what the direction of the weight vector is. Thus, the filtered Hebbian plasticity rule (together with the normalization rule not specified here) optimizes slowness (Equation 13) under the constraint of unit variance (Equation 6). Figure 2 illustrates this learning scheme. It also underlines the necessity for a clear distinction between processing and learning. Although the slowness principle does not allow low-pass filtering as a means of generating slow signals during processing, the learning rule may well make use of low-pass filtered signals to detect slowly varying features in the input signal. This distinction will become particularly important for the Poisson model neuron below, as it incorporates an excitatory postsynaptic potential (EPSP) that acts as a low-pass filter during processing. An implementation of the slowness principle in such a system must avoid the system exploiting the EPSP as a means of generating slow signals.

#### Alternative filtering procedures.

If learning is slow, the total weight change over a time interval [*t_a_*,*t_b_*] in a synapse can be written as

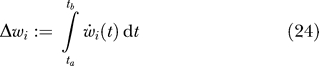


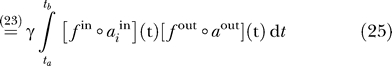


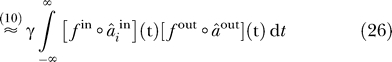


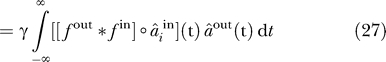


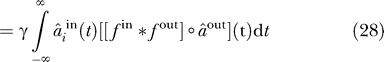


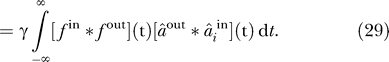



Thus, one can either convolve input and output signal with filters *f* ^in^ and *f* ^out^, respectively, the input signal with *f* ^out^ * *f* ^in^, or the output signal with *f* ^in^ * *f* ^out^. Note that [*f* ^in^ * *f* ^out^](t) = [*f* ^out^ * *f* ^in^](−*t*). One can actually use any pair of filters *f* ^in^ and *f* ^out^ as long as *f* ^in^ * *f* ^out^ fulfills the condition

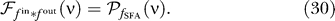



#### Relation to other learning rules.

Hebbian learning on low-pass filtered signals is the basis of several other models for unsupervised learning of invariances [1,4,6]. These models essentially subject the output signal to an exponential temporal filter *f*(*t*) = *θ*(*t)*exp(−*γt*) and then use Hebbian learning to associate it with the input signal. Here, *θ*(*t*) denotes the Heaviside step function, which is 0 for *t* < 0 and 1 for *t* ≥ 0. This learning rule has been named the “trace rule.” The considerations in the last section provide a link between this approach and ours. We simply have to replace *f* ^in^ with a *δ-*function and *f* ^out^ with *f*(*t*). Equation 29 then takes the form

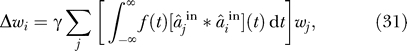
since the output signal 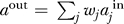 is a linear function of the input (see Equation 1). In the previously mentioned applications of the trace rule, the statistics of the input signals were always reversible, so we will assume that all correlation functions 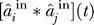 are symmetric in time. This implies that only the symmetric component of *f*(*t*) is relevant for learning:

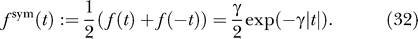
It is easy to show that the learning rule in Equation 31 can be interpreted as a gradient ascent on the following objective function:

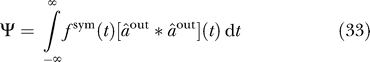


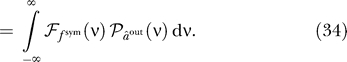
By comparison with Equation 19, it becomes clear that the trace rule implements a very similar objective as our model. The only difference is that the power spectrum in Equation 20 is replaced by the Fourier transform of the filter *f* ^sym^. Note that in order to be able to interpret Ψ as an objective function, it should be real-valued. The replacement of *f* with *f* ^sym^ ensures that 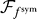 is real-valued and symmetric, so Ψ is real-valued as well. The Fourier transform of *f* ^sym^ is given by

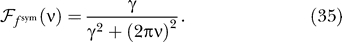
This shows that the only difference between the trace rule and our model lies in the choice of the power spectrum for the low-pass filter. While we are using a parabolic power spectrum with a cutoff (Equation 20), the trace rule uses a power spectrum with the shape of a Cauchy function (Equation 35).

From this perspective, one can interpret SFA as a quadratic approximation of the trace rule. To what extent this approximation is valid depends on the power spectra of the input signals. If most of the input power is concentrated at low frequencies, where the power spectrum resembles a parabola, the learning rules can be expected to learn very similar weight vectors. In fact, any Hebbian learning rule that leads to an objective function of the shape of Equation 19 with a low-pass filtering spectrum in the place of 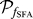 essentially implements the slowness principle, as among signals with the same variance, it will favor slower ones.

### Spiking Model Neuron

Real neurons do not transmit information via a continuous stream of analog values like the model neuron considered in the previous section, but rather emit action potentials that carry information by means of their rate and probably also by their exact timing, a fact we will not consider here. How can the model developed so far be mapped onto this scenario?

#### The linear Poisson neuron.

Again, we restrict our analysis to a simple case by modeling the spike-train signals by inhomogeneous Poisson processes. Note that at this point, we restrict our analysis to a rate code, thus neglecting possible coding paradigms that rely on precise timing of spikes.

To generate the input spike trains, we first add sufficiently large constants 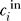 to the continuous and zero-mean signals 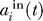 to turn them into strictly positive signals that can be interpreted as rates

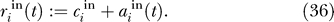



The constants 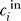 represent mean firing rates, which are modulated by the input signals 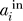 . From the input rates 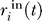 , we then derive inhomogeneous Poisson spike trains 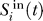 drawn from ensembles 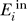 such that

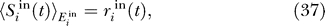
where 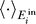 denotes the average over the ensemble 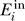 .

The output rate is modeled as a weighted sum over the input spike trains convolved with an EPSP *ɛ*(*t*) plus a baseline firing rate *r*_0_, which ensures that the output firing rate remains positive. This is necessary as we allow inhibitory synapses (i.e., negative weights).

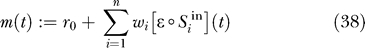



Note that in this scheme, the EPSP reflects the change in the postsynaptic firing probability due to a presynaptic spike rather than a change in the membrane potential. Ideally, it includes all delay effects in neuronal transmission.

The output of this spiking neuron is yet another inhomogeneous Poisson spike train *S* ^out^(*t*) drawn from an ensemble *E* ^out^, given a realization of the input spike trains 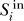 such that

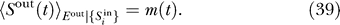



It should be noted that not only is the output spike train *S* ^out^(*t*) stochastic in this model, but also the underlying output rate *m*(*t*), which is a function of the stochastic variables 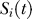 and generally differs for each realization of the input. This is the reason why the input and output spike trains are not statistically independent. However, due to the linearity of the model neuron, the output rate is still simply

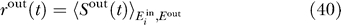


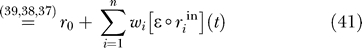


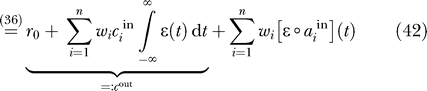


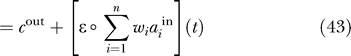


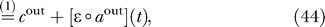
and the joint firing rate is

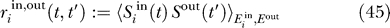


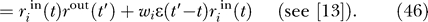



The first term would result also from a rate model, while the second term captures the statistical dependencies between input and output spike trains mediated by the synaptic weights *w_i_* and the EPSP *ɛ*(*t*).

#### STDP can perform SFA.

In this section, we will demonstrate that in an ensemble-averaged sense it is possible to generate the same weight distribution as in the continuous model by means of an STDP rule with a specific learning window.

Synaptic plasticity that depends on the temporal order of pre- and postsynaptic spikes has been found in a number of neuronal systems [14–18], and has raised a lot of interest among modelers [19,20] (for a review, see [21]). Typically, synapses undergo long-term potentiation (LTP) if a presynaptic spike precedes a postsynaptic spike within a timescale of tens of milliseconds and long-term depression (LTD) for the opposite temporal order. Assuming that the change in synaptic efficacy occurs on a slower timescale than the typical interspike interval, the STDP weight dynamics can be modeled as

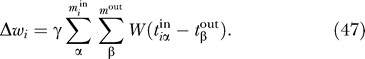



Here, 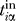 denotes the spike times of the presynaptic spikes at synapse *i* and 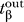 denotes the postsynaptic spike times. *W*(*t*) is the learning window that determines if and to what extent the synapse is potentiated or depressed by a single spike pair. The convention is such that negative arguments *t* in *W*(*t*) correspond to the situation where the presynaptic spike precedes the postsynaptic spike. 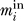 and *m* ^out^ are the numbers of pre- and postsynaptic spikes occurring in the time interval [*t_a_*, *t_b_*] under consideration. *γ* is a small positive learning rate. Note that due to the presence of this learning rate, the absolute scale of the learning window *W* is not important for our analysis.

We circumvent the well-known stability problem of STDP by applying an explicit weight normalization (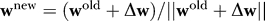 ) instead of weight-dependent learning rates as used elsewhere [22–24]. Such a normalization procedure could be implemented by means of a homeostatic mechanism targeting the output firing rate (e.g., by synaptic scaling; for reviews, see [25,26]).

Modeling the spike trains as sums of delta pulses (i.e., 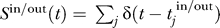 ), the learning rule in Equation 47 can be rewritten as

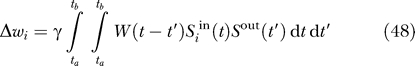


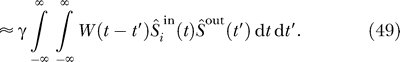



Taking the ensemble average allows us to retrieve the rates that underlie the spike trains and thus the signals 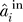 and 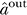 of the continuous model:

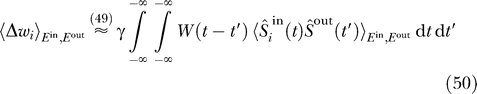


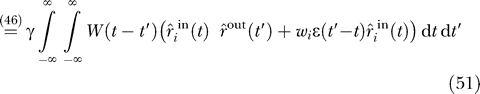


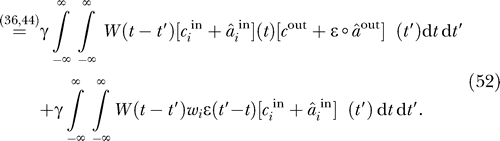



Expanding the products in Equation 52 gives rise to a number of terms, among which only one depends on both the input and the output signal 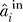 and 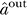 . Because each input signal has a vanishing mean, terms containing just one input signal lead to negligible contributions. The remaining terms depend only on the mean firing rates 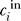 and 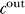 :

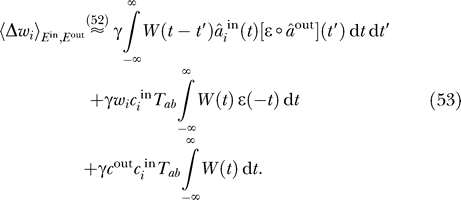



A generalized version of Equation 53 that incorporates non-Hebbian plasticity (i.e., terms that depend on the pre/postsynaptic signals only) has been derived and discussed by Kempter et al. [27]. Regarding the effects of the input signals on learning, the decisive term is the first one. The other two are rather unspecific in that they do not depend on the properties of the input and output signals 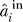 and 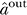 .

The second term alone would generate a competition between the weights: synapses that experience a higher mean input firing rate 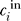 grow more rapidly than those with smaller input firing rates. If we assume that the input neurons fire with the same mean firing rate, all weights grow with the same rate, so the direction of the weight vector remains unchanged. Thus, due to the explicit weight normalization, this term has no effect on the weight dynamics and can be neglected.

If the integral over the learning window is positive, the third term in Equation 53 favors a weight vector that is proportional to the vector of the mean firing rates of the input neurons. It thus stabilizes the homogeneous weight distribution and opposes the effect of the first term, which captures correlations in the input signals. Note that this is only true if the integral over the learning window is positive; otherwise, this term introduces a competition between the weights [24,27]. One possible interpretation is that the neuron has a “default state” in which all synapses are equally strong and that correlations in the input need to surpass a certain threshold in order to be imprinted in the synaptic connections. Interestingly, this threshold is determined by the integral over the learning window, which implies that neurons that balance LTP and LTD should be more sensitive to input correlations.

An alternative possibility is that the neuron possesses a mechanism of canceling the effects of this term. From a computational perspective this would be sensible, as the mean firing rates 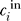 and 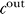 do not carry information about the input, neither in rate nor in a timing code. If we conceive neurons as information encoders aiming at adapting to the structure of their input, this term is thus more hindrance than help. Assuming that the neuron compensates for this term, the dynamics of the synaptic weights are governed exclusively by the correlations in the input signals as reflected by the first term. In the following, we will restrict our considerations to this term and omit the others.

Rearranging the temporal integrations, we can rewrite Equation 53 for the weight updates as

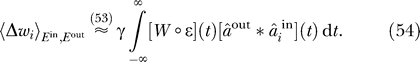



The first conclusion we can draw from this reformulation is that for the dynamics of the learning process the convolution of the learning window with the EPSP and not the learning window alone is relevant. As discussed below, this might have important consequences for functional interpretations of the shape of the learning window.

Second, by comparison with Equation 29, it is obvious that in order to learn the same weight distribution as in the continuous model, the learning window has to fulfill the condition that

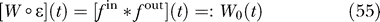


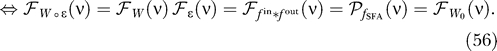



Here, *W*_0_ is the convolution of *W* with *ɛ* and is equal to the learning window in the limit of an infinitely short, *δ*-shaped EPSP. As the power spectrum 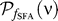 is of course real, *W*_0_ is symmetric in time. Note that the width of *W*_0_ scales inversely with the width of the power spectrum 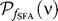 , which in turn is proportional to ν_max_. Once the power spectrum 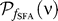 and the EPSP is given, Equation 56 uniquely determines the learning window *W*. Because it is *W*_0_ rather than *W* that determines the learning dynamics, we will refer to *W*_0_ as the “effective learning window.”

#### Learning windows.

According to the last section, we require special learning windows to learn the slow directions in the input. This of course raises the question of which window shapes are favorable, and in particular if these are in agreement with physiological findings.

Given the shape of the EPSP and the power spectrum 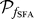 , the learning window is uniquely determined by Equation 56. Remember that the only parameter in the power spectrum 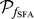 is the frequency *ν*_max_, above which the power spectrum of the input data was assumed to vanish. For simplicity, we model the EPSP as a single exponential with a time constant *τ*:

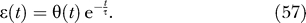



For this particular EPSP shape, the learning window can be calculated analytically by inverting the Fourier transform in Equation 56. The result can be written as

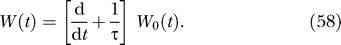

*W*_0_ is symmetric, so its derivative is antisymmetric. Thus, the learning window is a linear combination of a symmetric and an antisymmetric component. As the width of *W*_0_ scales with the inverse of *ν*_max_, its temporal derivative scales with *ν*_max_. Accordingly, the symmetry of the learning window is governed by an interplay of the duration *τ* of the EPSP and the maximal input frequency *ν*_max_. For *τ* ≪ 1 / *ν*_max_ the learning window is dominated by *W*_0_ and thus symmetric, whereas for *τ* ≫ 1 / *ν*_max_, the temporal derivative of *W*_0_ is dominant, so the learning window is antisymmetric.

We have assumed that the input signals have negligible power above the maximal input frequency *ν*_max_. Thus, the temporal structure of the input signals can only provide a lower bound for *ν*_max_. On the other hand, exceedingly high values for *ν*_max_ lead to very narrow learning windows, thereby sharpening the coincidence detection and reducing the speed of learning. Moreover, it may be metabolically costly to implement physiological processes that are faster than necessary. Thus, it appears sensible to choose *ν*_max_ such that 1 / *ν*_max_ reflects the fastest timescale in the input signals. Accordingly, the symmetry of the learning window is governed by the relation between the length of the EPSP and the fastest timescale in the input data. If the EPSP is short enough to resolve the fastest input components, the learning window is symmetric. If the EPSP is too long to fully resolve the temporal structure of the input (i.e., it acts as a low-pass filter), the learning window will tend to be antisymmetric.

We choose a value of *ν*_max_ = 1 / (40 ms). The argument for this choice is that within a rate code, the cells that project to the neuron under consideration can hardly convey signals that vary on a faster timescale than the duration of their EPSP. It is thus reasonable to choose the time constant of the EPSP and the inverse of the cutoff frequency to have the same order of magnitude. Typical durations of cortical EPSPs are of the order of tens of milliseconds (see [28] for further references and a critical discussion), so 40 ms seems a reasonable value.

**Figure 3:**
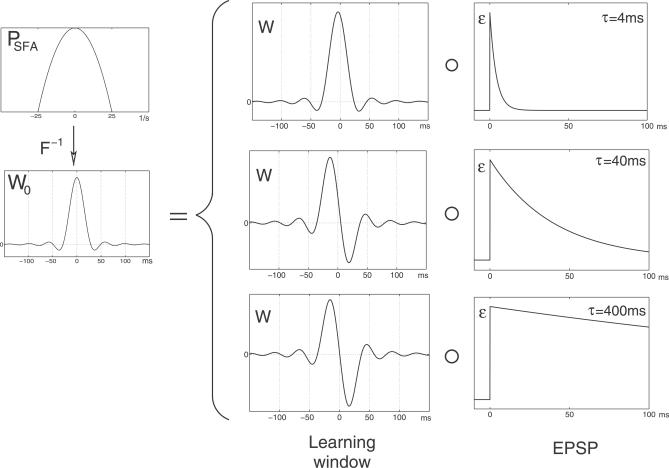
Relation between the EPSP and the Learning Window The power spectrum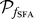 is the Fourier transform of the effective learning window *W*_0_, which in turn is the convolution of the learning window *W* and the EPSP *ɛ*. The figure shows the learning windows required for SFA for three different EPSP durations (*τ* = 4, 40, 400 ms). The maximal input frequency *ν*_max_ was 1 / (40 ms) in all plots.

Figure 3 illustrates the connection between 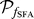 , *W*_0_, the learning window, and the EPSP. It also shows the learning windows for three different durations of the EPSP, while keeping *ν*_max_ = 1 / (40 ms). The oscillatory and slowly decaying tails of *W*(*t*) are due to the sharp cutoff of the power spectrum 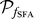 at |*ν*| = *ν*_max_ and become less pronounced if 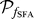 is smoothened out.

**Figure 4:**
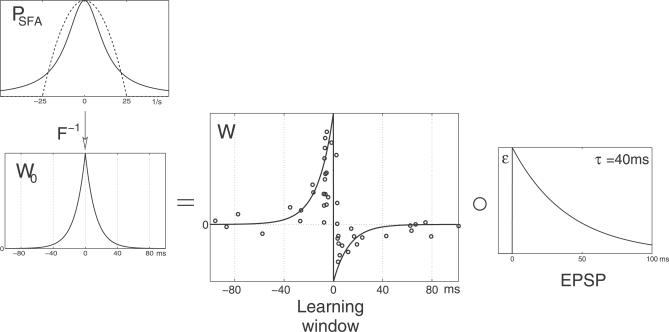
Comparison of the Learning Window with Experimental Data The plot compares the theoretically predicted learning window with experimental data from hippocampal pyramidal cells as published by Bi and Poo [16] (larger plot in the middle). Instead of the ideal power spectrum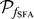 with the abrupt cutoff at *ν*_max_ as stated in Equation 20, a Cauchy function with *γ* = 1 / 15 ms was used (top left, the dashed line is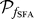 for *ν*_max_ = 1 / (40 ms)). Again, the EPSP decay time was *τ* = 40 ms. This learning window corresponds to an implementation of the “trace rule” [1,4,6] for a decay time of the exponential filter of 15 ms.

As negative time arguments in *W*(*t*) correspond to the case in which the presynaptic spike (and thus the onset of the resulting EPSP) precedes the postsynaptic spike, the shape of the theoretically derived learning window for physiologically plausible values of *τ* and *ν*_max_ (*τ* = 1 / *ν*_max_ = 40 ms; middle row in Figure 3) predicts potentiation of the synapse when a postsynaptic spike is preceded by the onset of an EPSP and depression of the synapse when this temporal order is reversed. This behavior is in agreement with experimental data from neocortex and hippocampus in rats as well as from the optic tectum in *Xenopus* [14–18]. To further illustrate this agreement, Figure 4 compares the data as published by Bi and Poo [16] with the learning window resulting from a smoothened power spectrum with the shape of a Cauchy function (Equation 35) instead of 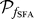 . As demonstrated above, this corresponds to implementing the slowness principle in form of the trace rule. Interestingly, the resulting learning window has the double-exponential shape that is regularly used in models of STDP (e.g., [24,29,30]). As the absolute scale of the learning window is not determined in our analysis, it was adjusted to facilitate the comparison with the experimental data.

#### Interpretation of the learning windows.

The last section leaves a central question open: why are these learning windows optimal for slowness learning and why does the EPSP play such an important role for the shape of the learning window?

Let us first discuss the case of the symmetric learning window, that is, the situation in which the EPSP is shorter than the fastest timescale in the input signal. Then, the convolution with the EPSP has practically no effect on the temporal structure of the signal and the output firing rate can be regarded as an instantaneous function of the input rates. We can thus neglect the EPSP altogether. The learning mechanism can then be understood as follows: assume at a given time *t* the postsynaptic firing rate *r* ^out^ is high and causes a postsynaptic spike. Then, the finite width of the learning window leads to potentiation not only of those synapses that participated in initiating the spike but also of those that transmit a spike within a certain time window around the time of the postsynaptic spike. As this leads to an increase of the firing rate within this time window, the learning mechanism tends to equilibrate the firing rates for neighboring times and thus favors temporally slow output signals.

If the duration of the EPSP is longer than the fastest timescale in the input signal, the output firing rate is no longer an instantaneous function of the input signals but generated by low-pass filtering the signal *a* ^out^ with the EPSP. This affects learning, because the objective of the continuous model is to optimize the slowness of *a* ^out^, whose temporal structure is now “obscured” by the EPSP. In order to optimize the objective, the system thus has to develop a deconvolution mechanism to reconstruct *a* ^out^. From this point of view, the learning window has to perform two tasks simultaneously. It has to first perform the deconvolution and then enforce slowness on the resulting signal. This is most easily illustrated by means of the condition in Equation 55. The convolution of the learning window with the EPSP generates the effective learning window *W*_0_ that is independent of the EPSP and which coincides with the learning window for infinitely short EPSPs. Intuitively, we could solve Equation 55 by choosing a learning window that consists of the “inverse” of the EPSP and the EPSP-free learning window *W*_0_. An intuitive example is the limiting case of an infinitely long EPSP. The EPSP then corresponds to a Heaviside function and performs an integration, which can be inverted by taking the derivative. Thus, the learning window for long EPSPs is the temporal derivative of the learning window for short EPSPs. The dependence of the required learning window on the shape of the EPSP is thus caused by the need of the learning window to “invert” the EPSP.

These considerations shed a different light on the shape of physiologically measured learning windows. The antisymmetry of the learning window may not act as a physiological implementation of a causality detector after all, but rather as a mechanism for compensating intrinsic low-pass filters in neuronal processing such as the EPSP. For functional interpretations of STDP, it may be more sensible to consider the convolution of the learning window with the EPSP than the learning window alone.

It should be noted that, according to our learning rule, the weights adapt in order to make a hypothetical instantaneous output signal *a* ^out^ optimally slow. This does not necessarily imply that the output firing rate *r* ^out^, which is generated by low-pass filtering *a* ^out^ with the EPSP, is optimally slow. In principle, the system could generate more slowly varying signals by exploiting the temporal structure of the EPSP. However, the motivation for the slowness principle is the idea that the system learns to detect invariances in the *input* signal, and that from this perspective the goal of creating a slowly varying output signal is not an end in itself but a means to learn invariances. Thus, the low-pass filtering effect of the EPSP should not be exploited but ignored or compensated.

#### General learning windows and EPSPs.

Although the asymmetry in LTP/LTD induction observed by Bi and Poo [16] has also been observed in other studies, the decay times for the LTP and the LTD branches of the learning window appear to be different in other preparations [18]. One may thus ask how robust our interpretation is with respect to the detailed shape of the learning window. To address this question, we start with some general learning window *W* and EPSP *ɛ* and ask under which conditions the effective learning window *W*_0_ = *W*_°_ *ɛ* prefers slowly varying features in the input.

As a starting point, we use the dynamics of the weights in Equation 54 as generated by the input statistics. Using 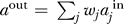 and defining the correlation functions 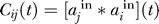 yields

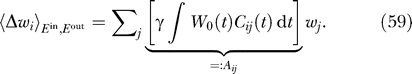
The dynamics thus follows a linear difference equation with a dynamic matrix *A_ij_* whose properties are determined by the correlation function *C_ij_*(*t*) and the effective learning window *W*_0_(*t*). One important question is whether the weights approach a stable fixed-point state or oscillate. In this context, the symmetry properties of *A_ij_* and thus those of *C_ij_* are crucial. The correlation functions obey the relation

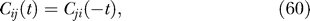
which couples their spatial symmetry (i.e., the symmetry with respect to the indices *i* and *j*) to their temporal symmetry. For instance, if the input statistics are reversible, i.e., for *C_ij_*(*t*) = *C_ij_*(−*t*), *C_ij_* is symmetric in the indices and so is *A_ij_*. If the input statistics were “perfectly irreversible,” i.e., *C_ij_*(*t*) = − *C_ij_*(−*t*), *C_ij_* and *A_ij_* would be antisymmetric. This motivates the splitting of the correlation functions *C_ij_* into a temporally symmetric and an antisymmetric component: *C_ij_* = *C_ij_^+^ + *C_ij_^−^** with *C_ij_^±^(t)* = ±*C_ij_^±^(−t)*. In a similar fashion, we split the effective learning window *W*_0_ = *W*_0_^+^ + *W*_0_^−^. For symmetry reasons, the dynamical matrix *A_ij_* can then be separated into two components

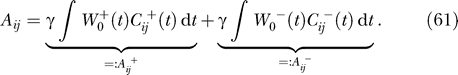



Because of the symmetry relation in Equation 60, *A_ij_* ^+^ is symmetric in *i* and *j*, while *A_ij_* ^−^ is antisymmetric. This shows that the effective learning window *W*_0_ can be split into two functionally different components. The symmetric component picks up the reversible aspects of the input statistics while the antisymmetric component detects irreversibilities, e.g., possible causal relations within the input data. It is this antisymmetric component of the learning window that has previously been interpreted as a means for sequence learning and predictive coding [19,31]. Note that the associated weight update ∑*_j_A_ij_^−^w_j_* is always orthogonal to the weight itself. Thus, irreversibilities in the input data in combination with an antisymmetric learning window work against the development of a stable weight distribution, even if the input statistics are stationary. In particular, weight oscillations on the timescale of learning may occur. For instance, in networks with recurrent connections that learn according to STDP, previous studies have shown that the network tends to develop a state of distributed synchrony [32] that resembles synfire chains. These activity patterns display a pronounced causal structure, so it would be interesting to check if the synaptic weights that emerge in such a network are stable or show oscillations. It is likely that in this context the model constraints on the weights play an important role. If the weights are limited by hard boundaries as in [32], they tend to saturate, thereby avoiding oscillatory solutions. In the case of softer weight constraints, e.g., in models of STDP with multiplicative weight-dependence, oscillations may occur.

If *W*_0_ is symmetric or if the input statistics are reversible, *C_ij_^−^* = 0, the dynamical matrix *A_ij_* = *A_ij_* ^+^ is symmetric. As already seen for the case of the continuous model neuron, the learning dynamics can then be interpreted as a gradient ascent on the objective function

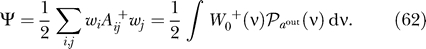



As discussed earlier, this objective function can be interpreted as an implementation of the slowness principle if *W*_0_^+^(ν) is a low-pass filter, i.e., it has a global maximum at zero frequency. This indicates that at least for reversible input statistics the preference of STDP for slow signals may be rather insensitive to details of the learning window.

## Discussion

Neurons in the central nervous system display a wide range of invariances in their response behavior, examples of which are phase invariance in complex cells in the early visual system [33], head direction invariance in hippocampal place cells [34], or more complex invariances in neurons associated with face recognition [35]. If these invariances are learned, the associated learning rule must somehow reflect a heuristics as to which sensory stimuli are supposed to be categorized as being the same. Objects in our environment are unlikely to change completely from one moment to the next but rather undergo typical transformations. Intuitively, responses of neurons with invariances to these transformations should thus vary more slowly than others. The slowness principle uses this intuition and conjectures that neurons learn these invariances by favoring slowly varying output signals without exploiting low-pass filtering.

SFA [10] is one implementation of the slowness principle in that it minimizes the mean square of the temporal derivative of the output signal for a given set of training data. SFA has been used to model a wide range of physiologically observed properties of complex cells in primary visual cortex [8] as well as translation, rotation, and other invariances in the visual system [10]. In combination with a sparse coding objective, SFA has also been used to describe the self-organized formation of place cells in the hippocampal formation [11].

The algorithm that underlies SFA is rather technical, and it has not yet been examined whether it is feasible to implement SFA within the limitations of neuronal circuitry. In this paper we approach this question analytically and demonstrate that such an implementation is possible in both continuous and spiking model neurons.

In the first part of the paper, we show that for linear continuous model neurons, the slowest direction in the input signal can be learned by means of Hebbian learning on low-pass filtered versions of the input and the output signal. The power spectrum of the low-pass filter required for implementing SFA can be derived from the learning objective and has the shape of an upside-down parabola.

The idea of using low-pass filtered signals for invariance learning is a feature that our model has in common with several others [1,4,6]. By means of the continuous model neuron, we have discussed the relation of our model to these “trace rules” and have shown that they bear strong similarities.

The second part of the paper discusses the modifications that have to be made to adjust the learning rule for a Poisson neuron. We find that in an ensemble-averaged sense it is possible to reproduce the behavior of the continuous model neuron by means of spike-timing–dependent plasticity (STDP). Our study suggests that the outcome of STDP learning is not governed by the learning window alone but rather by the convolution of the learning window with the EPSP, which is of relevance for functional interpretations of STDP.

The learning window that realizes SFA can be calculated analytically. Its shape is determined by the interplay of the duration of the EPSP and the maximal input frequency *ν*_max_, above which the input signals are assumed to have negligible power. If *ν*_max_ is small, i.e., if the EPSP is sufficiently short to temporally resolve the most quickly varying components of the input data, the learning window is symmetric, whereas for large *ν*_max_ or long EPSPs, it is antisymmetric. Interestingly, physiologically plausible parameters lead to a learning window whose shape and width is in agreement with experimental findings. Based on this result, we propose a new functional interpretation of the STDP learning window as an implementation of the slowness principle that compensates for neuronal low-pass filters such as the EPSP.

An important question in this context is on which timescales is this interpretation valid. It is conceivable that for signals that vary on a timescale of less than a hundred milliseconds, a learning window with a width of tens of milliseconds can distinguish slower from faster signals. STDP could thus be sufficient to establish invariant representations in early sensory processing, e.g., visual receptive fields that become invariant to microsaccades inducing small translations. Although it is unlikely that STDP alone can distinguish between signals that vary on behavioral timescales of hundreds of milliseconds or even seconds, this may not be problematic, because it is probably not sensible to order *all* aspects of the stimuli according to how quickly they vary. Rather, one should distinguish input components that vary so quickly that they are unlikely to be behaviorally relevant from those that vary on behavioral timescales. From this perspective, the intrinsic timescale of the learning rule should be such that its discriminative power is best on a timescale where this transition occurs. It is conceivable that this transition timescale lies on the order of several tens of milliseconds. The learning of high level invariances that correspond to behavioral timescales will probably require additional mechanisms with corresponding intrinsic timescales, e.g., sustained firing in response to a stimulus [36].

For general learning windows and EPSPs, the convolution of the learning window with the EPSP can be split into a symmetric component and an antisymmetric component. The symmetric component picks up reversible aspects of the input statistics while the antisymmetric component detects irreversible aspects. Previous functional interpretations of STDP have mostly concentrated on the antisymmetric component, which has been interpreted, e.g., as a mechanism for sequence learning or predictive coding [19,31] or for reducing recurrent connectivity in favor of feed-forward structures [30,32]. Other studies have neglected the phase structure of the learning window altogether and concentrated on its power spectrum, proposing that timing-dependent plasticity performs Hebbian learning on an optimal estimate of the input signals in the presence of noise [37,38]. Note that these interpretations are not necessarily contradictory to ours, because the slowness interpretation relies on the symmetric component of the learning window only and thus on the reversible aspect of the input statistics. These considerations indicate that depending on the temporal structure of the input, STDP may have different functional roles.

A different approach to unsupervised learning of invariances with a biologically realistic model neuron has been taken by Körding and König [39]. In their model, bursts of backpropagating spikes gate synaptic plasticity by providing sufficient amounts of dendritic depolarization. These bursts are assumed to be triggered by lateral connections that evoke calcium spikes in the apical dendrites of cortical pyramidal cells.

Of course the model presented here is not a complete implementation of SFA. We have only considered the central step of SFA, the extraction of the most slowly varying direction from a set of whitened input signals. To implement the full algorithm, additional steps are necessary: a nonlinear expansion of the input space, the whitening of the expanded input signals, and a means of normalizing the weights. When traversing the dendritic arborizations of a postsynaptic neuron, axons often make more than one synaptic contact. As different input channels may be subjected to different nonlinearities in the dendritic tree (cf. [40]), the postsynaptic neuron may have access to several nonlinearly transformed versions of the same presynaptic signals. Conceptually, this resembles a nonlinear expansion of the input signals. However, it is not obvious how these signals could be whitened within the dendrite. On the network level, however, whitening could be achieved by adaptive recurrent inhibition between the neurons [41]. This mechanism may also be suitable for extracting several slow uncorrelated signals as required in the original formulation of SFA [10] instead of just one. We assumed an explicit weight normalization in the description of our model. However, one could also use a modified learning rule that implicitly normalizes the weight vector as long as it extracts the signal with the largest variance. A possible biological mechanism is synaptic scaling [25], which is believed to multiplicatively rescale all synaptic weights according to postsynaptic activity, similar to Oja's rule [26,42]. Thus, it appears that most of the mechanisms necessary for an implementation of the full SFA algorithm are available, but that it is not yet clear how to combine them in a biologically plausible way.

Another critical point in the analytical derivation for the spiking model is the replacement of the temporal by the ensemble average, as this allows recovery of the rates that underlie the Poisson processes. The validity of the analytical results thus requires some kind of ergodicity in the training data, a condition which of course needs to be justified for the specific input data at hand.

It is still open whether the results presented here can be reproduced with more realistic model neurons. The spiking model neuron used here was simplified in that it had a linear relationship between input and output firing rate. In many real neurons, highly nonlinear behavior was observed. Interestingly, Hebbian learning for nonlinear rate-based neurons has previously been associated with the detection of higher-order moments of the input statistics [43], thereby providing a mechanism for extracting statistically independent components of the input signal. Because for sparse input statistics independent component analysis is closely related to sparse coding [44], it is tempting to speculate that within a rate picture, temporally nonlocal plasticity with a nonlinear input–output relation implements a combination of sparseness and slowness. Learning paradigms that combine these two objectives are thus an interesting field for further studies [11,45].

Another nonlinearity that we have neglected is the frequency- and weight-dependence of STDP [16,46]. Additional work will be needed to examine how these interfere with the proposed functional role of STDP. Furthermore, modeling the spiking mechanism of a neuron by an inhomogeneous Poisson process is also a severe simplification that ignores basic phenomena of spike generation in biological neurons such as refractoriness and thresholding. It is not clear how these characteristics would change the learning rule that leads to an implementation of the slowness principle. It seems to be a very difficult task to answer these questions analytically. Simulations will be necessary to verify the results derived here and to analyze which changes appear and which adaptations must be made in a more realistic model of neural information processing.

In summary, the analytical considerations presented here show that (i) slowness can be equivalently achieved by minimizing the variance of the time derivative signal or by maximizing the variance of the low-pass filtered signal, the latter of which can be achieved by standard Hebbian learning on the low-pass filtered input and output signals; (ii) the difference between SFA and the trace learning rule lies in the exact shape of the effective low-pass filter—for most practical purposes the results are probably equivalent; (iii) for a spiking Poisson model neuron with an STDP learning rule, it is not the learning window that governs the weight dynamics but the convolution of the learning window with the EPSP; (iv) the STDP learning window that implements the slowness objective is in good agreement with learning windows found experimentally. With these results, we have reduced the gap between slowness as an abstract learning principle and biologically plausible STDP learning rules, and we offer a completely new interpretation of the standard STDP learning window.

## Methods

The methods employed in this paper rely on standard mathematical techniques as commonly used in the theory of synaptic plasticity (see, e.g., [47]).

## Abbreviations

EPSP: excitatory postsynaptic potential
LTD: long-term depression
LTP: long-term potentiation
SFA: slow feature analysis
STDP: spike-timing–dependent plasticity
